# Older Adults as Citizen Scientists and Policy and Planning Voices in Higher Education

**DOI:** 10.1093/geroni/igaa057.1786

**Published:** 2020-12-16

**Authors:** Lenard Kaye, Sarah Burby

**Affiliations:** 1 University of Maine, Bangor, Maine, United States; 2 University of Maine School of Social Work, Orono, Maine, United States

## Abstract

The University of Maine is embarking on achieving AFU status. In addition to maximizing older adult participation in all facets of campus life (education, recreation, culture, etc.), their presence in nontraditional sectors of university activity will be emphasized. Building on the principles of community-based, participatory research, focal points of UMaine’s AFU strategy will be to ensure that age-specific, engagement mechanisms are created and maintained that ensure older citizens play an influential role in guiding and interpreting academic research and development and curricula innovation across multiple professions and disciplines. Using a state-wide, older adult research registry, and co-design, community test-beds in partnership with continuing care retirement communities, older adults will serve as citizen scientists. Other empowerment strategies for maximizing elder voice include expanding the number of departments that incorporate life span perspectives in their mission statements and expanding the number of older adult advisory bodies that inform university policy and practice.

